# Impact of the expression of thymidylate synthase and dihydropyrimidine dehydrogenase genes on survival in stage II/III gastric cancer

**DOI:** 10.1007/s10120-014-0413-8

**Published:** 2014-08-12

**Authors:** Mitsuru Sasako, Masanori Terashima, Wataru Ichikawa, Atsushi Ochiai, Koji Kitada, Issei Kurahashi, Shinichi Sakuramoto, Hitoshi Katai, Takeshi Sano, Hiroshi Imamura

**Affiliations:** 1 Department of Surgery, Hyogo College of Medicine, 1-1 Mukogawa-cho, Nishinomiya, Hyogo 663-8501 Japan; 2 Division of Gastric Surgery, Shizuoka Cancer Center, 1007 Shimonagakubo, Nagaizumi-cho, Sunto-gun, Shizuoka 411-8777 Japan; 3 Division of Medical Oncology, Department of Medicine, Showa University, School of Medicine, 1-5-8 Hatanodai, Shinagawa, Tokyo 142-8555 Japan; 4 Pathology Division, Research Center for Innovative Oncology, National Cancer Center Hospital East, 6-5-1 Kashiwanoha, Kashiwa, Chiba 277-8577 Japan; 5 Department of Surgery, National Hospital Organization, Fukuyama Medical Center, 4-14-17 Okinogami-cho, Fukuyama, Hiroshima 720-8520 Japan; 6 Department of Medical Informatics and Economics, The University of Tokyo, 7-3-1 Hongo, Bunkyo, Tokyo 113-0033 Japan; 7 Department of Surgery, Saitama Medical University, International Medical Center, 1397-1 Yamane, Hidaka, Saitama 350-1298 Japan; 8 Gastric Surgery Division, National Cancer Center Hospital, 5-1-1 Tsukiji, Chuo, Tokyo 104-0045 Japan; 9 Department of Gastroenterological Surgery, Cancer Institute Hospital, Japanese Foundation for Cancer Research, 3-8-31 Ariake, Koto, Tokyo 135-8550 Japan; 10 Department of Surgery, Toyonaka Municipal Hospital, 4-14-1 Shibahara-cho, Toyonaka, Osaka 560-8565 Japan

**Keywords:** Stomach neoplasms, Thymidylate synthase, Dihydrouracil dehydrogenase, Chemotherapy, adjuvant, Biological markers

## Abstract

**Background:**

The efficacy of 5-fluorouracil (5FU)-based therapy, which remains the cornerstone of gastrointestinal cancer treatment, depends upon the expression of enzymes involved in pyrimidine metabolism, including thymidylate synthase (TS), dihydropyrimidine dehydrogenase (DPD), thymidine phosphorylase (TP), and orotate phosphoribosyltransferase (OPRT). We analyzed the expression of these genes in patients enrolled in the Adjuvant Chemotherapy Trial of S-1 for Gastric Cancer (ACTS-GC) and their possible roles as biomarkers for treatment outcomes.

**Methods:**

Formalin-fixed, paraffin-embedded specimens were available for 829 of a total of 1,059 (78.3 %) patients. TS, DPD, TP, and OPRT expression was measured by RT-PCR in manually microdissected tumor specimens and normalized to the reference gene, β-actin. The expression level of each gene was categorized as low or high using cutoffs at the 33.3rd, 50th, or 66.7th percentiles.

**Results:**

The hazard ratio (HR) for overall survival (OS) after S-1 treatment versus surgery alone was significantly lower in high (>66.7th percentile; HR = 0.370; 95 % CI 0.221–0.619) compared to low (<66.7th percentile; HR = 0.757; 95 % CI 0.563–1.018) TS expression groups (*P* = 0.015). Similarly, the HR for OS after S-1 therapy versus surgery alone was significantly lower in high (>33.3rd percentile; HR = 0.520, 95 % CI 0.376–0.720) compared to low (<33.3rd percentile; HR = 0.848, 95 % CI 0.563–1.276) DPD expression groups (*P* = 0.065). There was no interaction between TP or OPRT expression and OS.

**Conclusions:**

This large biomarker study showed that high TS and DPD gene expression in tumors was associated with enhanced benefit from postoperative adjuvant S-1 treatment in gastric cancer. There was no interaction between TP and OPRT expression and S-1 treatment.

**Electronic supplementary material:**

The online version of this article (doi:10.1007/s10120-014-0413-8) contains supplementary material, which is available to authorized users.

## Introduction

Gastric cancer is the second commonest cause of cancer-related death worldwide. The mainstay of treatment for gastric cancer is surgery. However, in stage II (excluding T1) and stage III (moderately advanced) disease, many patients develop recurrence, even after curative resection. S-1 (TS-1; Taiho Pharmaceutical, Tokyo, Japan) is an oral fluoropyrimidine preparation combining tegafur, gimeracil, and oteracil potassium [1]. The Adjuvant Chemotherapy Trial of S-1 for Gastric Cancer (ACTS-GC), a prospective randomized phase III trial, demonstrated that surgery plus S-1 treatment was more effective than surgery alone in Japanese patients with stage II/III gastric cancer [2, 3]. However, the 5-year overall survival (OS) rate in patients with stage IIIB disease was 50.2 % in the S-1 group in a subset analysis, suggesting room for improvement [3]. Therefore, it is important to also evaluate the effectiveness of intensive preoperative and/or postoperative chemotherapy with multiple agents in patients at high risk of relapse. Alternatively, reliable biomarkers are needed to improve outcomes by enabling the selection of patients who would benefit from S-1 or other novel therapies. We previously reported that EGFR positivity, but not HER2 positivity, was associated with poor patient outcomes after curative resection of stage II/III gastric cancer, using archived specimens obtained from patients enrolled in the ACTS-GC [4]. Furthermore, there was no apparent interaction between S-1 and EGFR or HER2 status with respect to survival [4].

Several enzymes play key roles in fluoropyrimidine metabolism. Thymidylate synthase (TS) is the rate-limiting enzyme in the de novo synthesis of 2′-deoxy-thymidine-5′-monophosphate, which is required for DNA synthesis and repair, and is therefore the primary target of fluoropyrimidines [5]. Dihydropyrimidine dehydrogenase (DPD) is the rate-limiting enzyme in 5-fluorouracil (5FU) catabolism [6]. Thymidine phosphorylase (TP) and orotate phosphoribosyltransferase (OPRT) convert 5FU to active metabolites such as 2′-deoxy-5-fluorouridine and 5-fluorouridine-5′-monophosphate, respectively [7]. Basically, high TS, DPD, and TP expression and low OPRT expression in tumors have been thought to result in relatively low sensitivity to fluoropyrimidine-based chemotherapy [5–8]. Many studies have evaluated correlations between the expression levels of these enzymes and clinical outcomes using gastrointestinal tumor specimens, suggesting that the expression of them could allow the accurate prediction of clinical outcome in patients receiving fluoropyrimidine-based chemotherapy [9]. However, the clinical significance of the expression of these genes remains unclear, as many inconsistent results are reported in the literature, and most published reports concern retrospective analyses of data from nonrandomized or relatively small randomized studies.

In this study we have therefore measured the expression of TS, DPD, TP, and OPRT genes by RT-PCR in gastric tumor specimens obtained from patients enrolled in the ACTS-GC. We evaluated them retrospectively to determine whether their expression levels would be predictive markers for a response to S-1 and/or prognostic markers.

## Materials and methods

### Study population and design

**Fig. 1:**
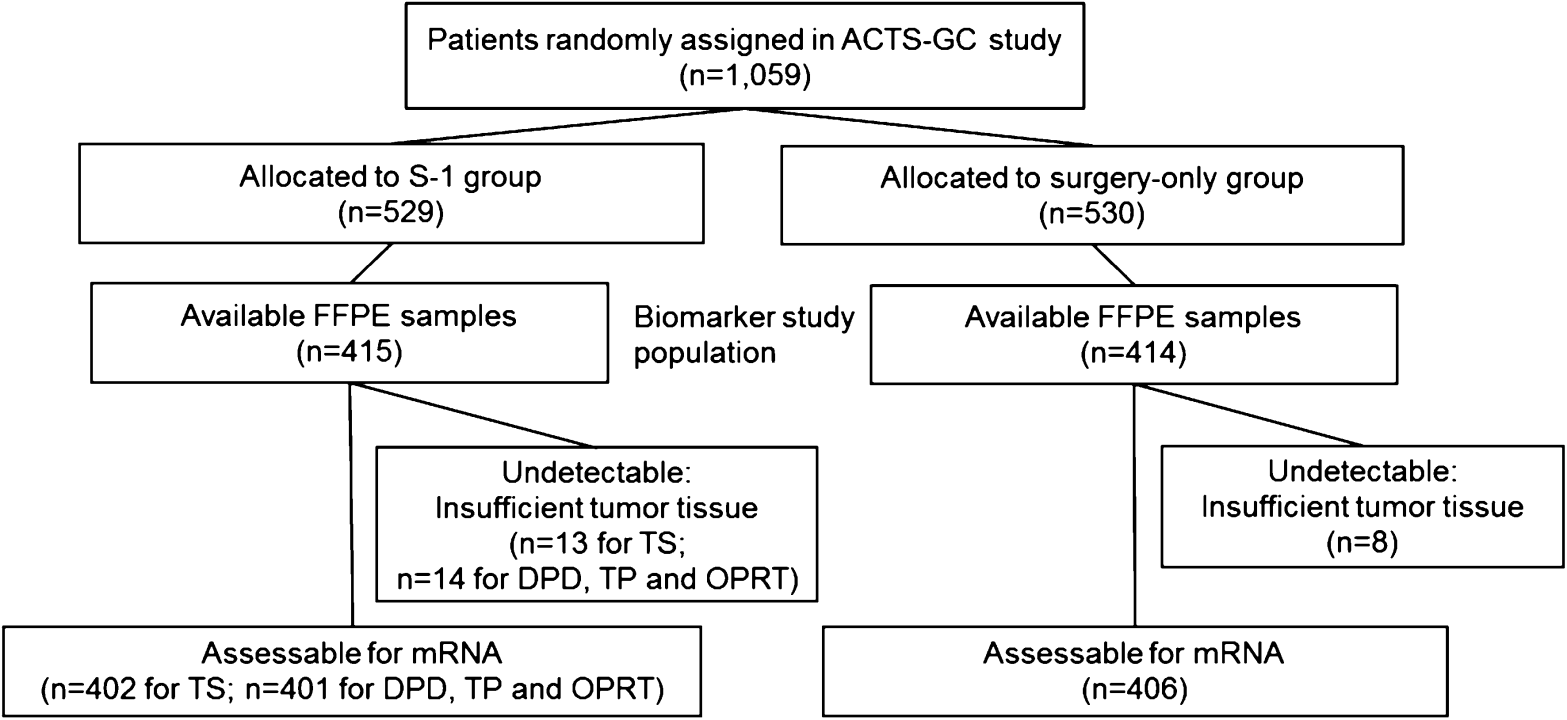
Diagram of patient flow

Tumor tissue was collected from patients enrolled in the ACTS-GC, for which the inclusion criteria and treatment protocol have been described previously [2, 3]. This biomarker study was designed retrospectively, after the completion of the first interim analysis of the ACTS-GC, to determine any predictive value for benefit from S-1 treatment or for prognosis [4]. Archived formalin-fixed, paraffin-embedded (FFPE) specimens obtained by surgical resection were available for 829 (78.3 %) of the 1,059 patients who were enrolled in the ACTS-GC at 65 centers and constituted the biomarker study population (Fig. 1). The protocol used for this biomarker study was approved by the ethics committee of the Japanese Gastric Cancer Association and the institutional review board of each participating hospital. This study also complied with REMARK guidelines [10], as shown in Table S1 of the Electronic supplementary material (ESM).

### Reverse transcription PCR

Representative hematoxylin and eosin stained slides from FFPE specimens were reviewed by a pathologist to estimate tumor load. Sections 10 μm in thickness were then stained with Nuclear Fast Red (Sigma–Aldrich, St Louis, MO, USA) for manual microdissection. Tumor tissue was selected at a magnification of 5–10× and dissected using a scalpel, as described previously [11].

RNA was isolated from tumor tissue and cDNA was prepared as described previously [12], with a slight modification to the extraction step, which used RNeasy MinElute spin columns (Qiagen, Chatsworth, GA, USA). Expression levels of the TS, DPD, TP, and OPRT genes were determined using TaqMan real-time PCR (Life Technologies, Foster City, CA, USA), as described previously [12]. β-Actin was used as an endogenous reference gene. The measurement of amplified cDNA used the cycle threshold (*Ct*) value, which is inversely proportional to the amount of cDNA. Gene expression values (relative mRNA levels) were expressed as ratios (differences between the *Ct* values) of the gene of interest (TS, DPD, TP, and OPRT) to a reference gene (β-actin). This reference gene provided a baseline measurement for the amount of mRNA isolated from a specimen. The expression levels of each gene were categorized as low or high at the 33.3rd, 50th, or 66.7th percentiles.

### Immunohistochemistry

All reagents and instruments for immunohistochemistry (IHC) were purchased from Ventana Medical Systems, Inc. (Tucson, AZ, USA). FFPE, 3–5 μm thick sections were automatically stained using a Ventana BenchMark^®^ ULTRA with primary monoclonal antibodies specific for TS, DPD, and TP and a polyclonal antibody specific for OPRT, prepared by Taiho [13–15], and an iView DAB Universal Kit, according to the manufacturer’s instructions. Staining was evaluated using light microscopy by two independent pathologists (KK and AO) who were blind to all clinical information. Tumor cell immunostaining was assessed semiquantitatively in three randomly selected × 20 fields in a semiquantitative manner to reflect both the intensity of staining and percentage of cells stained. Intensity was classified as unstained (0), weakly stained (1+), moderately stained (2+), or strongly stained (3+).

### Statistical analysis

Categorized data was analyzed using the chi-square test. Either the Wilcoxon test or the Kruskal–Wallis test was used to assess correlations between groups. Survival curves were estimated using the Kaplan–Meier product limit method, and the statistical significance of differences between survival curves was assessed using the log-rank test. Univariate and multivariate survival analyses were performed using a Cox proportional hazards model. Results were considered statistically significant at *P* < 0.05, except for the interaction test, for which *P* < 0.1 was considered statistically significant [16, 17]. Because this analysis was primarily exploratory, adjustments for multiple comparisons were not made [16]. All statistical analyses used the SAS software package (version 9.1) and JMP software (version 8.01; SAS Institute Inc., Cary, NC, USA).

We estimated the minimum difference in survival that would be required to show a significant survival difference between patients with tumors in which gene expression was high or low in each treatment arm. Each arm included approximately 400 patients. Given a tertile or median cutoff point, demonstrating a statistically significant difference in survival between patients with tumors with high and low gene expression would require HRs of at least 0.56 and 0.58, respectively, assuming a two-sided *α* = 0.05 and a power of 80 % in a proportional hazards model.

## Results

### Patient characteristics

There was no significant difference between the population used in this biomarker study and the total population of the ACTS-GC (Table 1), as previously reported [2]. The groups were well balanced with respect to gene expression levels and other factors.

**Table 1:** Patient characteristics

	Total ACTS-GC population			Biomarker study subpopulation		
	S-1 (*N* = 529)	Surgery only (*N* = 530)	*P* value^a^	S-1 (*N* = 415)	Surgery only (*N* = 414)	*P* value^a^
Sex, no. (%)						
Male	367 (69.4)	369 (69.6)	0.98	282 (68.0)	283 (68.4)	0.90
Female	162 (30.6)	161 (30.4)		133 (32.0)	131 (31.6)	
Age, no. (%)						
<60 years	199 (37.6)	195 (36.8)	0.86	160 (38.6)	158 (38.2)	0.72
60–69 years	193 (36.5)	215 (40.6)		149 (35.9)	161 (38.9)	
70–80 years	137 (25.9)	120 (22.6)		106 (25.5)	95 (22.9)	
Median (years)	63	63		63	62	
Range (years)	27–80	33–80		27–80	33–80	
Tumor stage, no. (%)						
T1	1 (0.2)	0 (0)	0.81	1 (0)	0 (0)	0.93
T2	289 (54.6)	286 (54.0)		222 (53.5)	223 (53.9)	
T3	225 (42.5)	232 (43.8)		180 (43.5)	182 (44.0)	
T4	14 (2.6)	12 (2.3)		12 (2.9)	9 (2.2)	
Nodal stage, no. (%)^b^						
N0	51 (9.6)	64 (12.1)	0.72	40 (9.6)	52 (12.6)	0.52
N1	296 (56.0)	281 (53.0)		233 (56.1)	222 (53.6)	
N2	182 (34.4)	185 (34.9)		142 (34.2)	140 (33.8)	
N3	0 (0)	0 (0)		0 (0)	0 (0)	
Lymph-node metastases, no. (%)						
0	51 (9.6)	64 (12.1)	0.37	40 (9.6)	52 (12.6)	0.18
1–6	331 (62.6)	325 (61.3)		254 (61.2)	254 (61.4)	
7–15	117 (22.1)	113 (21.3)		97 (23.4)	85 (20.5)	
≥16	30 (5.7)	28 (5.3)		24 (5.8)	23 (5.6)	
Cancer stage, no. (%)^c^						
II	236 (44.6)	238 (44.9)	0.78	183 (44.1)	189 (45.7)	0.48
IIIA	202 (38.2)	207 (39.1)		159 (38.3)	162 (39.1)	
IIIB	90 (17.0)	85 (16.0)		73 (17.6)	63 (15.2)	
IV	1 (0.2)	0 (0)		0 (0)	0 (0)	
Histologic type, no. (%)^d^						
Differentiated	214 (41.6)	209 (40.3)	0.73	166 (40.0)	166 (40.1)	0.91
Undifferentiated	301 (58.4)	307 (59.7)		249 (60.0)	245 (59.2)	
TS expression level, no. (%)^e^						
Low	–	–	–	138 (34.3)	134 (33.0)	0.72
Intermediate	–	–		137 (34.1)	131 (32.3)	
High	–	–		127 (31.6)	141 (34.7)	
DPD expression level, no. (%)^e^						
Low	–	–	–	136 (33.9)	133 (32.8)	0.60
Intermediate	–	–		135 (33.7)	135 (33.3)	
High	–	–		130 (32.4)	138 (34.0)	
TP expression level, no. (%)^e^						
Low	–	–	–	129 (32.2)	140 (34.5)	0.80
Intermediate	–	–		131 (32.7)	139 (34.2)	
High	–	–		141 (35.2)	127 (31.3)	
OPRT expression level, no. (%)^e^						
Low	–	–	–	129 (32.2)	140 (34.5)	0.23
Intermediate	–	–		131 (32.7)	140 (34.5)	
High	–	–		141 (35.2)	126 (31.0)	

Characteristics of all ACTS-GC patients can be found in the literature [2]

^a^ *P* values for sex and histologic type were calculated using the chi-square test. *P* values for age, tumor stage, nodal stage, number of lymph-node metastases, cancer stage (Japanese classifications), and gene expression level were calculated using the Wilcoxon test

^b^Nodal stages were defined according to the Japanese classification as follows: N0, no evidence of lymph-node metastasis; N1, metastasis to group 1 lymph nodes; N2, metastasis to group 2 lymph nodes; N3, metastasis to group 3 lymph nodes. Groups 1, 2, and 3 are regional lymph-node classifications defined according to the location of the primary tumor and based on the results of studies of lymphatic flow at various tumor sites and the observed survival associated with metastasis at each nodal station (i.e., position in relation to primary node)

^c^Cancer stages were defined according to the Japanese classification as follows: stage IA, T1N0; stage IB, T1N1 or T2N0; stage II, T1N2, T2N1, or T3N0; stage IIIA, T2N2, T3N1, or T4N0; stage IIIB, T3N2 or T4N1; stage IV, T4N2, any T stage with N3 or distant metastasis

^d^In the total ACTS-GC population, histologic type was classified for eligible patients (*N* = 1,034). In the surgery-only group of the biomarker study population, cancers could not be classified as differentiated or undifferentiated in three patients

^e^Gene expression levels were undetectable for some of the samples, as shown in Fig. 1

### Expression of TS, DPD, TP, and OPRT

Gene expression was assessable in 808 patients for TS and in 807 patients for DPD, TP, and OPRT, representing 97 % of the biomarker study population (Fig. 1). Histograms of the expression values for each gene showed typical normal distributions (see Fig. S1 of the ESM). Each relative mRNA level at the 33.3rd, 50th, and 66.7th percentile was as follows: 2.47, 3.03, and 3.87 for TS; 0.50, 0.69, and 0.97 for DPD; 4.19, 5.44, and 7.09 for TP; and 0.45, 0.54, and 0.67 for OPRT, respectively.

We classified patients into four groups according to TS, DPD, TP, and OPRT protein levels measured by IHC and scored as 0, 1+, 2+, and 3+. Representative examples of immunostaining for each gene product are shown in Fig. S2 of the ESM. IHC scores and gene expression levels for TS, TP, and OPRT were significantly correlated (*P* < 0.001), and there was considerable overlap between the four groups (see Fig. S3 of the ESM). On the other hand, IHC scores for DPD did not correlate with gene expression levels (*P* > 0.05), with more than half of the patients classified as 3+ by IHC.

### Correlation of the expression of *TS*, *DPD*, *TP*, and *OPRT* genes on survival

In the biomarker study population, 5-year OS and relapse-free survival (RFS) were 73.6 % [95 % confidence interval (CI) 69.3–77.9 %] and 66.7 % (95 % CI 62.1–71.3 %), respectively, in the S-1 group, compared with 61.9 % (95 % CI 57.1–66.7 %) and 53.7 % (95 % CI 48.8 %–58.7 %), respectively, in the surgery-only group. These figures were similar to the ACTS-GC 5-year follow-up data [3].

When gene expression was categorized as low or high using the 66.7th percentile, high TS expression was significantly associated with good OS and RFS in the S-1 group only (Table 2). In contrast, when gene expression was categorized as low or high using the 33.3rd percentile, high DPD expression was significantly associated with good OS and RFS in the S-1 group only (Table 3). There was no significant association of TS and DPD expression—categorized using the median—with outcomes in each group, although these figures were similar to the results obtained using the 66.7th and 33.3rd percentiles (data not shown).

**Table 2:** Univariate analysis of OS and RFS: expression of each gene was categorized as low or high at the 66.7th percentile

Marker	Group	Status	Number of patients	OS			RFS		
				5-Year survival (%)	HR (95 % CI)	*P* value (log-rank)	5-Year survival (%)	HR (95 % CI)	*P* value (log-rank)
TS	All	Low	540	66.5	1	0.222	58.1	1	0.085
		High	268	71.8	0.844 (0.642–1.109)		66.2	0.805 (0.629–1.031)	
	S-1	Low	275	69.9	1	0.008	62.2	1	0.003
		High	127	83.9	0.521 (0.319–0.850)		78.9	0.530 (0.344–0.816)	
	Surgery only	Low	265	63.0	1	0.623	53.8	1	0.923
		High	141	60.8	1.088 (0.777–1.522)		54.9	1.015 (0.747–1.380)	
DPD	All	Low	539	68.9	1	0.589	60.8	1	0.941
		High	268	66.9	1.075 (0.828–1.395)		60.6	1.009 (0.796–1.279)	
	S-1	Low	271	73.2	1	0.522	66.0	1	0.444
		High	130	76.3	0.870 (0.568–1.333)		70.1	0.862 (0.590–1.261)	
	Surgery only	Low	268	64.5	1	0.230	55.5	1	0.486
		High	138	58.0	1.225 (0.879–1.708)		51.6	1.114 (0.822–1.509)	
TP	All	Low	539	66.7	1	0.233	59.2	1	0.209
		High	268	71.1	0.848 (0.647–1.112)		63.8	0.856 (0.672–1.091)	
	S-1	Low	260	72.3	1	0.317	65.8	1	0.368
		High	141	77.6	0.806 (0.528–1.230)		70.2	0.843 (0.581–1.223)	
	Surgery only	Low	279	61.5	1	0.585	53.1	1	0.512
		High	127	63.8	0.907 (0.637–1.290)		56.6	0.898 (0.652–1.238)	
OPRT	All	Low	540	66.2	1	0.120	58.4	1	0.108
		High	267	72.2	0.805 (0.612–1.059)		65.7	0.818 (0.639–1.046)	
	S-1	Low	260	71.6	1	0.125	64.9	1	0.196
		High	141	78.9	0.715 (0.465–1.100)		72.0	0.779 (0.533–1.139)	
	Surgery only	Low	280	61.2	1	0.635	52.3	1	0.436
		High	126	64.7	0.918 (0.644–1.309)		58.7	0.879 (0.636–1.216)

**Table 3:** Univariate analysis of OS and RFS: expression of each gene was categorized as low or high at the 33.3rd percentile

Marker	Group	Status	Number of patients	OS			RFS		
				5 year survival (%)	HR (95 % CI)	*P* value (log-rank)	5 year survival (%)	HR (95 % CI)	*P* value (log-rank)
TS	All	Low	272	67.9	1	0.969	57.1	1	0.292
		High	536	68.4	0.995 (0.766–1.293)		62.7	0.883 (0.700–1.113)	
	S-1	Low	138	72.3	1	0.595	62.9	1	0.270
		High	264	75.3	0.897 (0.599–1.341)		70.0	0.819 (0.574–1.169)	
	Surgery only	Low	134	63.2	1	0.769	51.1	1	0.559
		High	272	61.8	1.053 (0.745–1.488)		55.7	0.913 (0.672–1.240)	
DPD	All	Low	269	64.7	1	0.137	57.9	1	0.180
		High	538	69.9	0.823 (0.636–1.064)		62.2	0.853 (0.676–1.076)	
	S-1	Low	136	66.8	1	0.015	60.8	1	0.039
		High	265	78.0	0.616 (0.416–0.914)		70.8	0.690 (0.485–0.983)	
	Surgery only	Low	133	62.6	1	0.942	55.1	1	0.978
		High	273	62.1	1.013 (0.718–1.429)		53.8	0.996 (0.730–1.359)	
TP	All	Low	269	64.4	1	0.148	56.8	1	0.168
		High	538	70.0	0.827 (0.640–1.070)		62.7	0.850 (0.673–1.072)	
	S-1	Low	129	67.7	1	0.067	62.0	1	0.116
		High	272	77.2	0.690 (0.463–1.029)		69.8	0.750 (0.523–1.075)	
	Surgery only	Low	140	61.5	1	0.831	52.0	1	0.776
		High	266	62.6	0.964 (0.688–1.351)		55.3	0.957 (0.706–1.296)	
OPRT	All	Low	269	67.1	1	0.838	57.0	1	0.246
		High	538	68.7	0.973 (0.749–1.264)		62.6	0.872 (0.691–1.099)	
	S-1	Low	129	74.3	1	0.907	66.4	1	0.877
		High	272	74.1	1.025 (0.674–1.559)		67.8	0.971 (0.671–1.406)	
	Surgery only	Low	140	60.5	1	0.807	48.4	1	0.191
		High	266	63.2	0.959 (0.685–1.342)		57.3	0.819 (0.608–1.105)	

There was no association between TP or OPRT expression and outcomes in either the S-1 or surgery-only groups (Tables 2, 3). Furthermore, there was no association between IHC scores for these four genes and outcomes (data not shown).

### Predictive value of biomarker analysis

**Fig. 2a–e:**
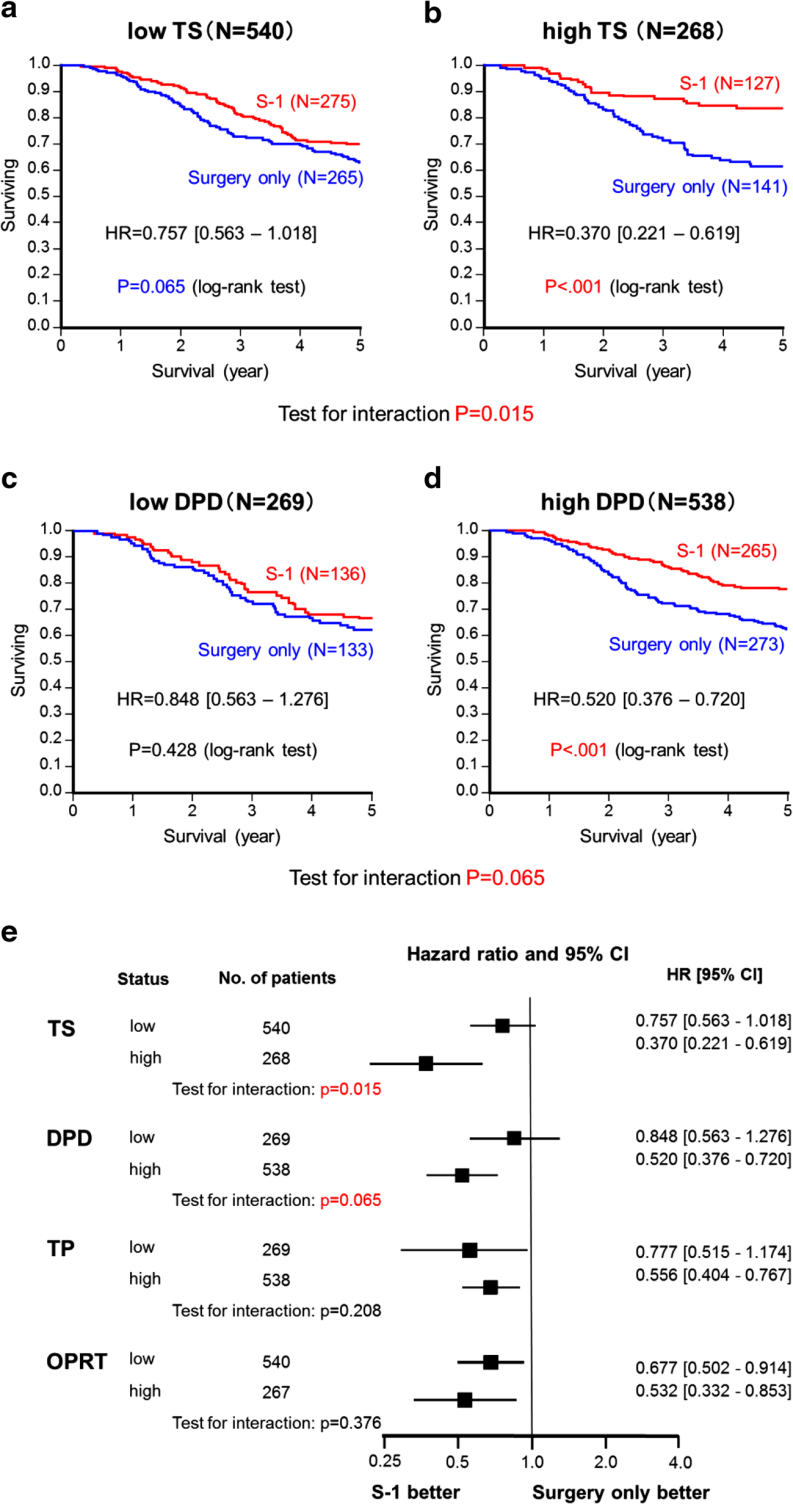
Kaplan–Meier curves showing overall survival for patients in the S-1-treated (*red*) and surgery-only (*blue*) groups for tumors with **a** low TS expression (<66.7th percentile), **b** high TS expression(>66.7th percentile), **c** low DPD expression (<33.3rd percentile), **d** high DPD expression (>33.3rd percentile). **e** Subgroup analysis of hazard ratios and overall survival

Kaplan–Meier plots of OS showed that S-1 treatment improved survival irrespective of TS or DPD expression (Fig. 2a–d). The HR for OS of the S-1 to surgery-only groups was lower in the high TS expressing population (>66.7th percentile; HR = 0.370; 95 % CI 0.221–0.619) than in the low TS expressing population (<66.7th percentile; HR = 0.757; 95 % CI 0.563–1.018). This interaction between TS expression and OS was statistically significant (*P* = 0.015). Similarly, the HR for OS of the S-1 to surgery only groups was lower in the high DPD expressing population (>33.3rd percentile; HR = 0.520; 95 % CI 0.376–0.720) than in the low DPD expressing group (<33.3rd percentile; HR = 0.848; 95 % CI 0.563–1.276). This interaction was also statistically significant (*P* = 0.065).

Analysis of OS in the biomarker study population found no interactions with gender, age, cancer stage, or histological type (data not shown), but did find an interaction with TS and DPD expression (Fig. 2e). No interaction was found between TP or OPRT expression and S-1 treatment (data not shown).

### Prognostic impact of TS and DPD

Since univariate analysis had shown a significant association between both high TS and high DPD expression and a good outcome in the S-1 group, we also assessed the prognostic relevance of TS and DPD using a multivariate proportional hazards model adjusted for age, cancer stage (Japanese Classification of Gastric Carcinoma, second English edition) [18], and histological type. We found that cancer stage and TS expression were independent prognostic factors (Table 4).

**Table 4:** Cox regression multivariate analysis of prognostic factors for OS in the S-1 group

Factor	Group	Number of patients	5-Year survival (%)	HR (95 % CI)	*P* value
Age	<60	157	76.6	1	
	60–69	146	78.1	1.288 (0.995–1.665)	
	70–80	98	64.5	1.659 (0.990–2.773)	0.055
Cancer stage (Japanese classification)	II	177	82.8	1	<0.001
	IIIa	153	72.5	1.746 (1.345–2.267)	
	IIIb	71	55.3	3.047 (1.809–5.141)	
Histologic type	Differentiated	242	76.2	1	0.250
	Undifferentiated^a^	159	71.0	1.283 (0.838–1.956)	
TS (66.7th percentile)	Low	275	69.9	1	0.011
	High	126	83.7	0.537 (0.317–0.87)	
DPD (33.3rd percentile)	Low	136	66.8	1	0.053
	High	265	78.0	0.663 (0.44–1.005)	

## Discussion

This study retrospectively evaluated the influence of TS, DPD, TP, and OPRT expression on the outcome for patients enrolled in the ACTS-GC. We found an association between high TS and high DPD expression, a positive prognosis in the S-1 group only, and an enhanced benefit from S-1 treatment. This was unexpected, as it contradicted many previous studies.

Many studies have evaluated the correlation between TS and DPD expression levels in tumors and clinical outcomes for gastrointestinal cancer patients [12, 19–23]. Ichikawa reviewed these studies for gastric cancer and noted that most had found that TS expression was a prognostic marker for survival regardless of whether therapy was given in an adjuvant or metastatic setting [9]. Similarly, high temporal DPD gene expression has been correlated with a lack of response to fluoropyrimidine-based therapy and an adverse outcome for gastric cancer patients in many studies [9]. The majority of published studies concern retrospective analyses of data derived from mainly nonrandomized and relatively small studies, often from a single institution, so they may have some limitations with respect to power and bias. We believe this ACTS-GC biomarker study overcomes these disadvantages, since the biomarker population used was representative of the total study population in terms of survival analysis and clinicopathological factors, and gene expression values were well balanced in each treatment group (Table 1).

However, we have to consider reasons for the difference between our results and previous reports. First, we discuss a methodological issue. Since no methodology has yet been validated for measuring TS and DPD, and only a few studies have compared IHC with RT-PCR, we used both methods. Although IHC scores for TS correlated with RT-PCR results, those for DPD did not (see Fig. S3 of the ESM). The gene expression of DPD had a greater variability among the cases with an IHC score of 3+ (*N* = 434), comprising the majority of cases. We also observed considerable overlap in gene expression between the four groups used to score TS expression in IHC, which may result from the heterogeneous immunostaining frequently seen in different randomly selected areas of slides. We consider RT-PCR to be a more quantifiable method than IHC, at least in this study, as almost all tumor cells in FFPE sections were dissected for RT-PCR.

A second issue is the cutoff value used for RT-PCR, as an optimal value has not yet been defined and the median has been used in several previous studies [19, 22]. We planned to use three cutoff points in this study, and the significant cutoff points were found to be different for TS and DPD. Furthermore, we explored this issue by analyzing the relationship between using different cutoff values for stratification and the *P* values from log-rank tests for TS and DPD gene expression. As shown in Fig. S4 of the ESM, the lowest *P* values were observed at the 66.7th percentile for TS but the 33.3rd for DPD in the S-1 group. This indicated that the tertile was the optimal cutoff value for TS and DPD gene expression in this cohort.

High TS and high DPD expression have been thought to result in lower sensitivity to 5FU-based chemotherapy. In contrast, Fujiwara et al. reported that S-1 showed better antitumor activity than 5FU in GT3TKB human gastric tumor xenografts with high TS and DPD activity [24]. In GT3TKB xenografts, the 5FU incorporated into RNA was significantly higher in the S-1 group than in the 5FU group. They speculated that the increase in the 5-fluoro-2′deoxyuridine-5′-monophosphate level was insufficient to enhance TS inhibition, and blocking of RNA function by the increased level of 5-fluorouridine-5′-triphosphate (another mechanism of action of 5FU) may have predominated. It was also suggested that a potent DPD inhibitor such as gimeracil could be used to circumvent the resistance to 5FU that occurs at high levels of DPD activity [24, 25]. The unexpected results observed in this study may be explained by noting that S-1 showed some effects not presented by other fluoropyrimidines.

For colorectal cancer, conflicting results have been published on TS expression in metastases versus primary tumors, and on the response to 5FU chemotherapy in advanced colorectal cancer versus the survival benefit of adjuvant 5FU therapy [26–28]. Kormann et al. reported that adjuvant 5FU chemotherapy prolonged the survival of patients with high TS mRNA levels, based on archival FFPE colorectal tumor tissue from 309 patients [28]. Their suggested explanation for their results was that the major effect of adjuvant therapy is the eradication of circulating cancer cells before they become established, and the milieu of circulating cells is clearly different from that of an established tumor in many respects. Thus, the mechanism by which S-1 suppresses recurrence after surgery could differ from the mechanism it uses to inhibit the growth of advanced tumors. Furthermore, gastric tumor tissue is known to be highly heterogeneous and complex. Therefore, a small tumor cell population (e.g., HER2-positive cells) could play an important role in tumor recurrence, and surrounding stromal cells that may have roles in tumor angiogenesis and immunity could also contribute to tumor recurrence [29–31]. To understand the roles of TS and DPD in the suppression of recurrence by S-1, their expression in both tumors and the surrounding normal cells in a micrometastatic tumor model needs to be investigated.

The most critical limitation of this study is that the results were obtained from a single cohort, even though the ACTS-GC was a large, randomized, phase III trial. To confirm the reproducibility of our results, further retrospective and prospective biomarker studies using FFPE samples from gastric cancer patients treated with adjuvant S-1 will be needed, using the same RT-PCR method and cutoff point.

Recently, the CLASSIC study—another prospective, randomized, phase III trial—demonstrated that adjuvant capecitabine plus oxaliplatin treatment after curative D2 gastrectomy was also more effective than surgery alone in East Asian patients with stage II/III gastric cancer [32]. A subgroup analysis suggested that adjuvant capecitabine and oxaliplatin was beneficial for all subgroups, although relatively high HR (0.90) was observed in node-negative patients. Adverse events were observed more frequently in the CLASSIC study than in the ACTS-GC study [2]. At present, we have two standard treatments for gastric cancer in Asia, and determining which patients would derive most benefit from these treatments remains a clinical problem for the future. The present study suggests that the tumoral expression levels of TS and DPD could provide useful information for selecting adjuvant treatment, either S-1 monotherapy or doublet treatment. Gastric tumors with high expression levels of TS or DPD are thought to be capable of responding to S-1 alone, whereas doublet treatment (such as capecitabine with oxaliplatin) would be required for patients with low tumoral expression levels of TS or DPD, since these individuals have a poor prognosis after S-1 treatment alone. Additionally, our results may provide some insight into the molecular characteristics of relapsed tumors after adjuvant S-1 treatment. As the majority would be expected to have relatively low TS and DPD expression, 5FU-based therapy would still benefit patients with relapsed tumors. Further understanding of the molecular biological and pathology of gastric cancer is needed to improve treatment for this disease.

In conclusion, this study provided evidence that high TS and DPD expression were associated with a positive prognosis in S-1 treated patients only, and with an enhanced benefit from S-1 therapy. Stratification by TS, DPD, TP, and OPRT gene expression levels did not suggest the existence of a subgroup of stage II/III gastric cancer patients who should not be offered adjuvant S-1 therapy.

### Electronic supplementary material

Below is the link to the electronic supplementary material.

Supplementary material 1 (PDF 526 kb)

Supplementary material 2 (PDF 120 kb)

## Appendix

The following Japanese institutions participated in this study as ACTS-GC Biomarker Study Group: Iwate Medical University Hospital (Morioka), Iwate Prefectural Central Hospital (Morioka), National Hospital Organization Sendai Medical Center (Sendai), Miyagi Prefectural Cancer Center (Natori), Yamagata Prefectural Central Hospital (Yamagata), Tsuboi Cancer Center Hospital (Koriyama), Niigata Cancer Center Hospital (Niigata), Gunma University Hospital (Maehashi), Gunma Prefectural Cancer Center (Ota), Tsuchiura Kyodo General Hospital (Tsuchiura), Tsukuba University Hospital (Tsukuba), Dokkyo Medical University Hospital (Mibu), Tochigi Cancer Center (Utsunomiya), Saitama Medical University Hospital (Moroyama, Hidaka), Chiba University Hospital (Chiba), National Cancer Center Hospital East (Kashiwa), Chiba Cancer Center (Chiba), National Center for Global Health and Medicine (Tokyo), Showa University Toyosu Hospital (Tokyo), National Cancer Center Hospital (Tokyo), Tokyo Metropolitan Bokutoh Hospital (Tokyo), Tokyo Metropolitan Cancer and Infectious Diseases Center Komagome Hospital (Tokyo), Cancer Institute Hospital (Tokyo), Nihon University Itabashi Hospital (Tokyo), Tokyo Metropolitan Tama Medical Center (Fuchu), Showa General Hospital (Kodaira), Kitasato University East Hospital (Sagamihara), Shizuoka General Hospital (Shizuoka), Fujieda Municipal General Hospital (Fujieda), Showa Inan General Hospital (Komagane), Gifu Municipal Hospital (Gifu), Ogaki Municipal Hospital (Ogaki), Social Insurance Chukyo Hospital (Nagoya), Aichi Cancer Center Hospital (Nagoya), National Hospital Organization Nagoya Medical Center (Nagoya), Aichi Cancer Center Aichi Hospital (Okazaki), Fukui-ken Saiseikai Hospital (Fukui), Fukui Red Cross Hospital (Fukui), Toyama Prefectural Central Hospital (Toyama), Kyoto Second Red Cross Hospital (Kyoto), NTT West Osaka Hospital (Osaka), Osaka City General Hospital (Osaka), National Hospital Organization Osaka National Hospital (Osaka), Osaka Medical Center for Cancer and Cardiovascular Diseases (Osaka), Sakai Municipal Hospital (Sakai), Kinki University Hospital (Osaka-Sayama), Hyogo Cancer Center (Akashi), Kansai Rosai Hospital (Amagasaki), Hiroshima University Hospital (Hiroshima), Hiroshima City Asa Hospital (Hiroshima), Hiroshima Red Cross Hospital and Atomic-Bomb Survivors Hospital (Hiroshima), Shimane Prefectural Central Hospital (Izumo), Tottori University Hospital (Yonago), Yamaguchi University Hospital (Ube), National Hospital Organization Shikoku Cancer Center (Matsuyama), National Kyushu Cancer Center (Fukuoka), National Hospital Organization Kyushu Medical Center (Fukuoka), Kitakyushu Municipal Medical Center (Kitakyusyu), Kokura Memorial Hospital (Kitakyushu), Social Insurance Tagawa Hospital (Tagawa), Saga Prefectural Hospital Koseikan (Saga), Sasebo City General Hospital (Sasebo), Oita Prefectural Hospital (Oita), Saiseikai Kumamoto Hospital (Kumamoto), Japanese Red Cross Kumamoto Hospital (Kumamoto).
