# Erratum to: Impact of the expression of thymidylate synthase and dihydropyrimidine dehydrogenase genes on survival in stage II/III gastric cancer

**DOI:** 10.1007/s10120-014-0428-1

**Published:** 2014-10-08

**Authors:** Mitsuru Sasako, Masanori Terashima, Wataru Ichikawa, Atsushi Ochiai, Koji Kitada, Issei Kurahashi, Shinichi Sakuramoto, Hitoshi Katai, Takeshi Sano, Hiroshi Imamura

**Affiliations:** 1 Department of Surgery, Hyogo College of Medicine, 1-1 Mukogawa-cho, Nishinomiya, Hyogo 663-8501 Japan; 2 Division of Gastric Surgery, Shizuoka Cancer Center, 1007 Shimonagakubo, Nagaizumi-cho, Sunto-gun, Shizuoka, 411-8777 Japan; 3 Division of Medical Oncology, Department of Medicine, Showa University, School of Medicine, 1-5-8 Hatanodai, Shinagawa, Tokyo 142-8555 Japan; 4 Pathology Division, Research Center for Innovative Oncology, National Cancer Center Hospital East, 6-5-1 Kashiwanoha, Kashiwa, Chiba 277-8577 Japan; 5 Department of Surgery, National Hospital Organization, Fukuyama Medical Center, 4-14-17 Okinogami-cho, Fukuyama, Hiroshima 720-8520 Japan; 6 Department of Medical Informatics and Economics, The University of Tokyo, 7-3-1 Hongo, Bunkyo, Tokyo, 113-0033 Japan; 7 Department of Surgery, Saitama Medical University, International Medical Center, 1397-1 Yamane, Hidaka, Saitama 350-1298 Japan; 8 Gastric Surgery Division, National Cancer Center Hospital, 5-1-1 Tsukiji, Chuo, Tokyo, 104-0045 Japan; 9 Department of Gastroenterological Surgery, Cancer Institute Hospital, Japanese Foundation for Cancer Research, 3-8-31 Ariake, Koto, Tokyo, 135-8550 Japan; 10 Department of Surgery, Toyonaka Municipal Hospital, 4-14-1 Shibahara-cho, Toyonaka, Osaka 560-8565 Japan

## Erratum to: Gastric Cancer DOI 10.1007/s10120-014-0413-8

Unfortunately, errors appeared in this article.

In the **Results** section, under the heading “Predictive value of biomarker analysis”, at the end of the final sentence in the second paragraph, the parenthetical expression “(data not shown)” should be deleted, as the data actually are shown.

Also, in Table 4, for the factor “Histological type” (in column 1), in column 2 (“Group”) the word “Undifferentiated” should appear first, above “Differentiated”, and the superscript “a” on “Undifferentiated” should be deleted.

We apologize for the occurrence of these errors.

