# Supplementary material for: Impact of the expression of thymidylate synthase and dihydropyrimidine dehydrogenase genes on survival in stage II/III gastric cancer
Source: Gastric Cancer. 2014 Aug 12;18(3):538–48. doi: 10.1007/s10120-014-0413-8 (PMC4511074; doi:10.1007/s10120-014-0413-8)
Supplement: Supplementary file 1 — Supplementary material 1 (PDF 526 kb) [file 10120_2014_413_MOESM1_ESM.pdf]

Supplementary Figure S1.

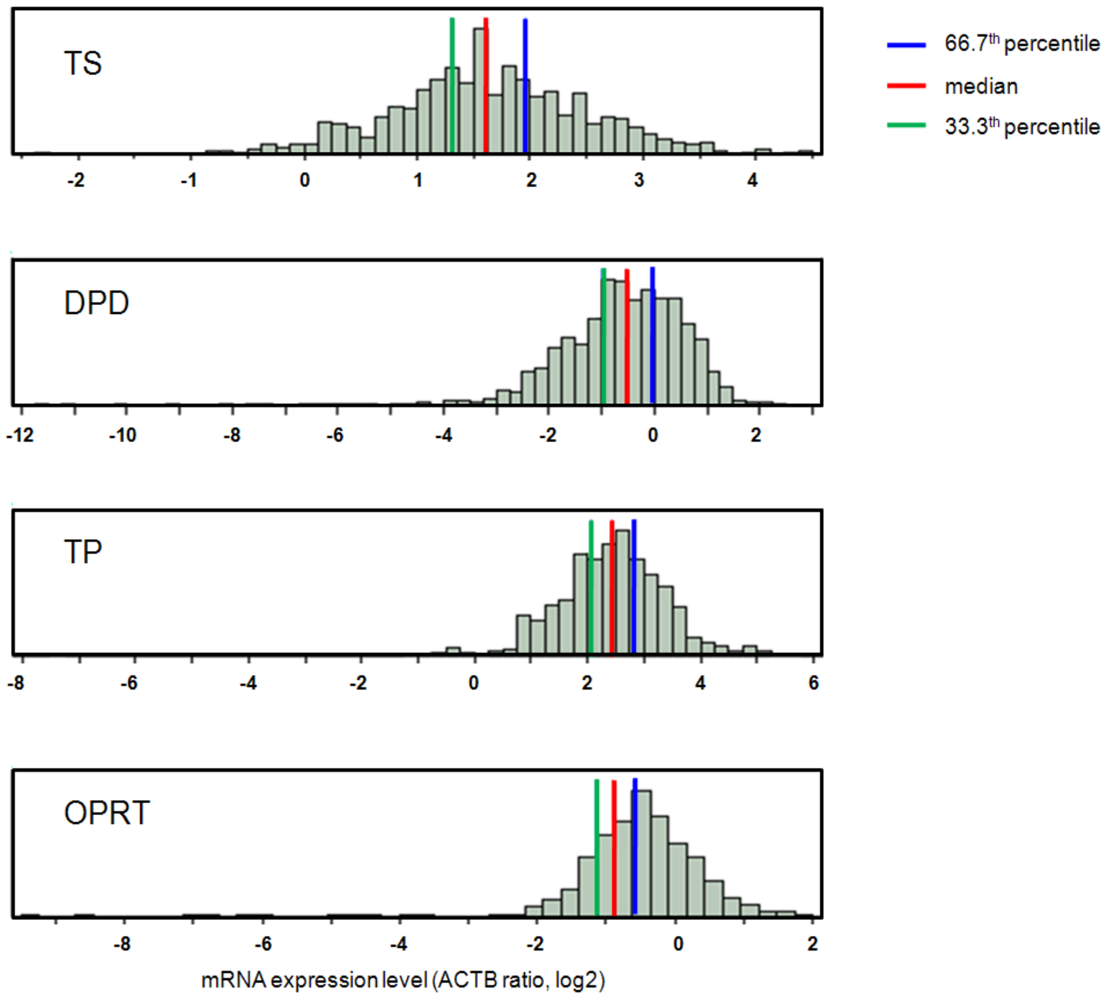

Supplementary Figure S1.
Histograms showing the distribution of TS, DPD, TP and OPRT gene expression measured by RT-PCR (x axis, expressed as log2). Each gene expression level at the 33.3rd, 50th, and 66.7th percentile was as follows: 2.47, 3.03, and 3.87 for TS; 0.50, 0.69, and 0.97 for DPD; 4.19, 5.44, and 7.09 for TP; and 0.45, 0.54, and 0.67 for OPRT, respectively.

Supplementary Figure S2.

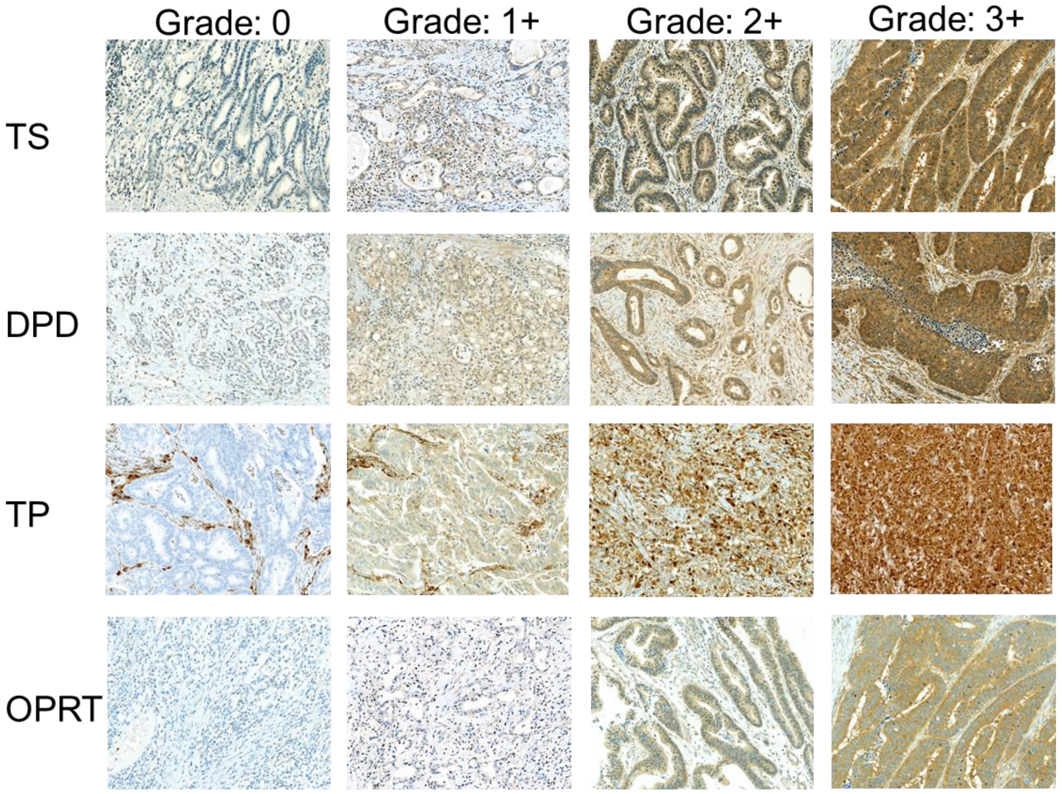

Supplementary Figure S2.
Representative examples of differently staged gastric carcinomas immunochemically stained for TS, DPD, TP and OPRT (magnification ×100).

Supplementary Figure S3.

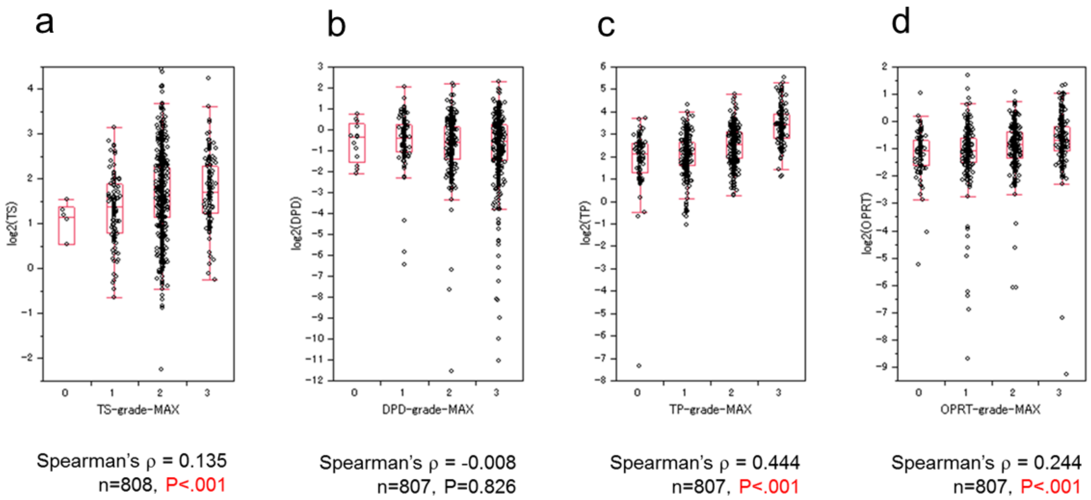

Supplementary Figure S3.
Relationship between TS, DPD, TP and OPRT expression levels in gastric tumors measured by RT-PCR (y axis, expressed as log2) and immunohistochemistry (IHC; x axis). a) Median TS mRNA levels for 4 IHC scores: 0, 1.15 (*N*=6); 1+, 1.38 (*N*=106); 2+, 1.61 (*N*=574); and 3+, 1.70 (*N* =122). b) Median DPD mRNA levels for 4 IHC scores: 0, -0.33 (*N* =13); 1+, -0.39 (*N* =102); 2+, -0.60 (*N* =258); and 3+, -0.53 (*N* =434). c) Median TP mRNA levels for 4 IHC scores: 0, 2.15 (*N* =91); 1+, 2.15 (*N* =290); 2+, 2.55 (*N* =295); and 3+, 3.43 (*N* =131). d) Median OPRT mRNA levels for 4 IHC scores: 0, -1.05 (*N* =86); 1+, -1.05 (*N* =218); 2+, -0.84 (*N* =271); and 3+, -0.68 (*N* =232). The Spearman's test was used to assess correlations between groups.
Box plots show the 25th (bottom) and 75th (top) percentiles, and the median (middle); the lowest datum was within 1.5 IQR of the lower quartile, and the highest datum was within 1.5 IQR of the upper quartile.

Supplementary Figure S4.

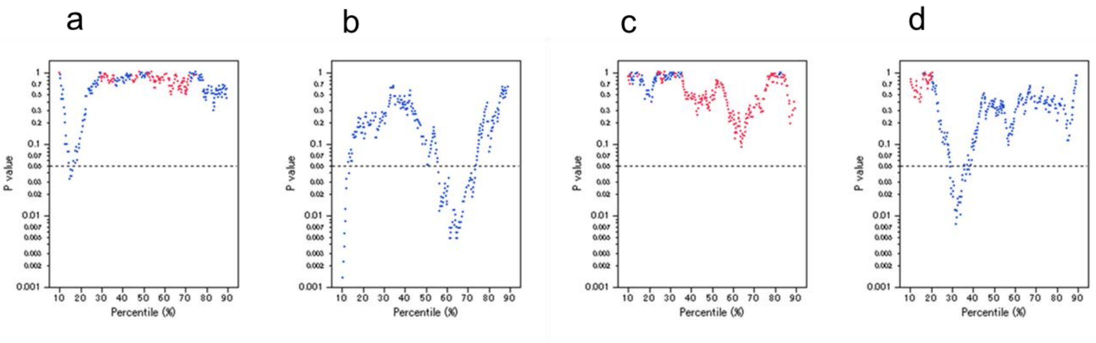

Supplementary Figure S4.

Correlations between cutoff values used for stratification and *P* values from log-rank tests for TS and DPD gene expression. a) *P* values for TS in the surgery-only group; b) *P* values for TS in the S-1-treated group; c) *P* values for DPD in the surgery-only group; d) *P* values for DPD in the S-1-treated group. Results below the dotted line (at $P<0.05$) are considered statistically significant.
