# Supplementary material for: Impact of the expression of thymidylate synthase and dihydropyrimidine dehydrogenase genes on survival in stage II/III gastric cancer
Source: Gastric Cancer. 2014 Aug 12;18(3):538–48. doi: 10.1007/s10120-014-0413-8 (PMC4511074; doi:10.1007/s10120-014-0413-8)
Supplement: Supplementary file 2 — Supplementary material 2 (PDF 120 kb) [file 10120_2014_413_MOESM2_ESM.pdf]

Supplementary Table S1. Compliance with REMARK guidelines

Introduction	
	Markers analyzed are enzymes involved in pyrimidine metabolism such as TS, DPD, TP and OPRT
	Objectives; to determine whether those markers are predictive for benefit from S-1 or prognostic
	Hypotheses; low TS and high OPRT expression are associated with enhanced benefit from S-1 and high TS, DPD and TP expression are associated with negative prognosis
Materials and Methods	
Patients	Patients with stage II/III gastric cancer randomized to adjuvant treatment with S-1 after surgery (D2 dissection) or surgery-only
Specimen characteristics	Formalin-fixed, paraffin-embedded surgical specimens
Assay method	Gene expression levels were determined by TaqMan real-time PCR in manual microdissected tumour specimens and normalized to β-actin expression as an endogenous reference gene
Study design	Retrospective analysis of paraffin-embedded material from patients treated within a prospective randomized study
	Clinical end-point of overall survival (OS) and relapse-free survival (RFS) used
	The expression levels of each gene were categorized as low or high at the 33.3rd, 50th or 66.7th percentile
	Sample size estimated by the availability of tissue from the 634 (60%) randomized patients enrolled in the ACTS-GC study; with the observed number of deaths power to detect interaction effects (ratio of treatment HRs) was ~80% for 0.5
Statistical methods	Categorical data analysis was conducted using either the chi-square test or the Wilcoxon test
	Univariate and multivariate analysis using Cox's proportional hazards regression model to assess the independent prognostic value of the expression levels of each gene

	Prognostic value of the expression levels of TS and DPD assessed in each treatment arm separately using Kaplan–Meier survival curves compared with the log-rank test
	Predictive value the expression levels of TS and DPD assessed using test for interaction of the subgroup treatment HRs, displayed in forest plots
Results	
Data	Flow of patients detailed in Figure 1; baseline characteristics of biomarker study sub-population for gene expression compared with total population of ACTS-GC in Table 1
Analysis and presentation	Distribution of the expression levels of each gene and association of them with IHC scores displayed in Supplementary Figures S1 and S3, available at online
	Representative examples of gastric carcinomas immunochemically stained for TS, DPD, TP and OPRT displayed in Supplementary Figures S2, available at online
	Univariate and multivariate analyses results displayed in Table 2-4
	Kaplan–Meier survival curves and forest plots for effect of TS and DPD on survival in Figure 2
	Correlations between cutoff values used for stratification and *P* values from log-rank tests for TS and DPD gene expression displayed in Supplementary Figures S4, available at online
Discussion	
	High TS and DPD expression were associated with positive prognosis only in S-1 group and enhanced benefit from S-1. Study limited by a single cohort analysis
